# Relationship between insulin resistance and amino acids in women and men

**DOI:** 10.14814/phy2.12392

**Published:** 2015-05-07

**Authors:** Ryan Seibert, Fahim Abbasi, Feras M Hantash, Michael P Caulfield, Gerald Reaven, Sun H Kim

**Affiliations:** 1 Department of Medicine, Stanford University School of MedicineStanford, California, USA; 2 Quest Diagnostics Nichols InstituteSan Juan Capistrano, California, USA

**Keywords:** Amino acids, insulin resistance, obesity, sex differences

## Abstract

Insulin resistance has been associated with higher plasma amino acid (AA) concentrations, but majority of studies have used indirect measures of insulin resistance. Our main objective was to define the relationship between plasma AA concentrations and a direct measure of insulin resistance in women and men. This was a cross-sectional study of 182 nondiabetic individuals (118 women and 64 men) who had measurement of 24 AAs and steady-state plasma glucose (SSPG) concentration (insulin resistance) using the insulin suppression test. Fourteen out of 24 AA concentrations were significantly (*P *< 0.05) higher in men than women; only glycine was lower in men. Majority of these AAs were positively associated with SSPG; only glycine concentration was negatively associated. Glutamic acid, isoleucine, leucine, and tyrosine concentrations had the strongest correlation with SSPG (*r* ≥ 0.4, *P *< 0.001). The degree of association was similar in women and men, independent of obesity, and similar to traditional markers of insulin resistance (e.g., glucose, triglyceride, high-density lipoprotein cholesterol). Compared with women, men tended to have a more unfavorable AA profile with higher concentration of AAs associated with insulin resistance and less glycine. However, the strength of association between a direct measurement of insulin resistance and AA concentrations were similar between sexes and equivalent to several traditional markers of insulin resistance.

## Introduction

Approximately 45 years ago, Felig, Marliss, and Cahill reported that branched chain amino acids (BCAA, leucine, isoleucine, valine) and aromatic amino acids (AAA, phenylalanine, tyrosine) were increased in 10 obese compared with 10 lean controls without diabetes (Felig et al. 1969). They also pointed out that insulin concentrations in response to a bolus of intravenous glucose were higher in obese compared with nonobese individuals, suggesting that “insulin ineffectiveness” was present in the obese individuals. Based on these findings they proposed that the hyperaminoacidemia in obesity may be secondary to insulin resistance.

The nature of the relationship between obesity, amino acids, and insulin resistance lay relatively dormant until the recent advent of metabolomics. Thus, Newgard et al. (2009) compared the results of comprehensive metabolic profiling of 74 obese and 67 lean individuals, and described increases in BCAA and AAA in obesity similar to the findings of Felig et al. (1969). These authors also found a relationship between estimates of insulin resistance and hyperaminoacidemia. More recently, studies in nonobese Chinese and Asian-Indian men (Tai et al. 2010) described a significant relationship between increases in plasma concentrations of a similar cluster of amino acids and insulin resistance “in individuals of relatively low body mass.”

Conclusions from these studies and more recent publications have all been based on surrogate estimates, not direct measurements of insulin resistance (Felig et al. 1969; Newgard et al. 2009; Tai et al. 2010; Würtz et al. 2012a, 2012b, 2013). Although correlated with direct measures of insulin resistance (Yeni-Komshian et al. 2000), surrogate markers also may lead to misleading results in certain populations, including nonobese groups (Kim et al. 2004) and mixed racial and sex groups (Pisprasert et al. 2013).

The major goal of this study was to define the relationship between 24 plasma amino acid concentrations and a direct measure of insulin-mediated glucose disposal in 182 nondiabetic individuals. In addition, we had the following three aims: (1) to evaluate the importance of possible sex differences in the relationship between amino acids and insulin resistance (Würtz et al. 2012a); (2) to see in a cross-sectional study if the relationship between insulin resistance and plasma amino acid concentrations was independent of variations in adiposity; and (3) to compare the magnitude of the relationship between plasma amino acid concentrations and a direct measure of insulin resistance with known insulin resistance–associated metabolic variables.

## Materials and Methods

### Subjects

The study population included 182 nondiabetic participants (118 women, 64 men) who had responded to advertisements describing our studies of glucose and insulin metabolism. All participants signed informed consent to participate in studies of insulin resistance, and were apparently healthy without history of coronary artery, kidney, or liver disease. Nondiabetic status was confirmed based on fasting glucose <126 mg/dL (American Diabetes Association 2014), no known medical history of diabetes, and no use of medications known to alter carbohydrate metabolism. They all had measurement of insulin resistance using the insulin suppression test.

### Measurements

#### Insulin suppression test

After an overnight fast for 12 h, all participants had a direct measurement of insulin resistance using the insulin suppression test (Pei et al. 1994) in the Stanford Clinical and Translational Research Unit. Values of insulin resistance using this technique are highly correlated (*r* ~ −0.9) with the hyperinsulinemic euglycemic clamp method (Greenfield et al. 1981; Knowles et al. 2013). After an overnight fast, an intravenous catheter was placed in each of the subject's arms. One arm was used for the administration of a 180-min infusion of octreotide (0.27 *μ*g/m^2^/min), insulin (32 mU/m^2^/min), and glucose (267 mg/m^2^/min); the other arm was used for collecting blood samples. Blood was drawn at 10-min intervals from 150 to 180 min of the infusion to determine steady-state plasma glucose (SSPG) and insulin concentrations. The SSPG concentration provides a direct measure of the ability of insulin to mediate disposal of an infused glucose load; therefore, the higher the SSPG concentration, the more insulin resistant is the individual.

#### Amino acid measurements

Plasma samples stored at −80°C from the morning of the insulin suppression test were analyzed for amino acids at Quest Diagnostics using liquid chromatography, mass spectrometry (LC/MS). Amino acids (24 total) from plasma sample were measured using an Agilent single quad LC/MS. The samples underwent protein precipitation then derivitized with phenylisothiocyanate (PITC). The samples were separated using gradient chromatography on a C-18 HPLC column and then identified by MS, where the mass/charge values corresponding to each particular amino acid is measured. The ratio of the area under the curves of each amino acid to its assigned internal standard was then plotted against a multiple point calibration curve, allowing for the quantitation of the amino acids in the patient samples. Intra-assay coefficient of variation (CV) for all amino acids ranged from 2% to 5% while the inter-assay CV was approximately 6%.

### Metabolic variables associated with insulin resistance

To compare the magnitude of the relationship between insulin resistance and amino acids, we assessed the relationship between SSPG and metabolic variables known to be associated with insulin resistance: fasting glucose, triglyceride, high-density lipoprotein cholesterol (HDL-C), and insulin concentrations (Kim and Reaven 2013). Triglyceride and HDL-C concentrations were measured in the core laboratory at Stanford University Medical Center. Glucose was determined by the oxidase method (Analyzer 2; Beckman, Brea, CA). Fasting plasma insulin concentrations were measured at Washington University (St. Louis, MO) using radioimmunoassay (Millipore, St. Charles, MO); the inter- and intra-assay CV ranged between 4.7% and 9.7%. Insulin concentrations were available on 114 individuals (75 women and 39 men).

### Statistical analysis

All statistical analyses were performed using SPSS (version 22 for Windows, SPSS Inc., Armonk, NY). Data are presented as mean ± SD or median [interquartile range]. Amino acids were log transformed prior to analysis. Pearson correlations (*r*-values and 95% confidence intervals) are reported between individual amino acids and SSPG concentration. Confidence intervals are based on bootstrapping with 1000 replications. To adjust for body mass index (BMI) or waist circumference, a linear regression model was used to predict SSPG with individual amino acids as the independent variable. Measurement of waist circumference was only available in 140 of 182 individuals. Amino acid concentrations were also compared between subjects in the lowest (most insulin sensitive) and highest (most insulin resistant) SSPG tertiles. Statistical significance was defined as *P *< 0.05.

## Results

Table1 presents demographic and baseline metabolic characteristics for the study population by sex. Women and men were not different in age, BMI, and degree of insulin resistance (SSPG). However, women had a better metabolic profile with lower fasting glucose and triglyceride concentration and higher HDL-C concentration. As expected, women also had lower creatinine concentration.

**Table 1:** Baseline characteristics of women and men

	Women	Men	*P*
	118	64	–
Age, years	52±9.5	52±7.2	0.79
Non-hispanic white, *n* (%)	81 (69%)	48 (75%)	0.39
BMI, kg/m^2^	29.6±5.2	29.9±4.2	0.67
Fasting plasma glucose, mg/dL	94.7±9.5	98.5±9.2	0.01
Triglyceride, mg/dL	130±96	171±100	0.001
HDL-C, mg/dL	52±15	43±10	<0.001
Creatinine, mg/dL	0.78±0.12	1.00±0.15	<0.001
Insulin, *μ*IU/mL	7.7±5.8	7.9±3.1	0.13
SSPG, mg/dL	144±72	161±64	0.11

Mean ± SD.

Sex-specific comparisons of amino acid concentrations are listed in Table2. These results demonstrate that concentrations were different as a function of sex in 15 of the 24 amino acids measured, with the values of men being significantly higher than in women in every instance with the exception of glycine.

**Table 2:** Comparison of AA concentrations (*μ*mol/L) between women and men

	Women	Men	*P*
Alanine	325 [278, 370]	330 [293, 374]	0.22
Arginine	71 [60, 82]	74 [61, 83]	0.64
Asparagine	32 [26, 42]	34 [27, 40]	0.43
Aspartic Acid	3 [3, 4]	4 [3, 5]	<0.001
Beta-Alanine	2 [2, 3]	3 [2, 3]	<0.001
Citrulline	29 [25, 36]	32 [26, 36]	0.02
Glutamic Acid	62 [44, 92]	87 [70, 118]	<0.001
Glutamine	541 [475, 594]	541 [498, 609]	0.20
Glycine	193 [170, 237]	182 [164, 205]	0.01
Histidine	67 [60, 74]	67 [60, 77]	0.64
Hydroxyproline	8 [6, 15]	9 [5, 17]	0.19
Isoleucine	56 [46, 6]	73 [63, 82]	<0.001
Leucine	106 [96, 120]	138 [122, 149]	<0.001
Lysine	158 [142, 177]	159 [143, 174]	0.35
Methionine	20 [18, 23]	23 [20, 25]	<0.001
Ornithine	50 [43, 59]	57 [48, 66]	0.005
Phenylalanine	56 [48, 62]	62 [55, 67]	0.001
Proline	147 [126, 173]	170 [142, 214]	<0.001
Sarcosine	2 [2, 3]	2 [2, 3]	0.003
Serine	83 [73, 97]	80 [69, 91]	0.34
Threonine	113 [95, 135]	109 [90, 118]	0.32
Tryptophan	44 [38, 50]	53 [49, 58]	<0.001
Tyrosine	58 [50, 66]	63 [59, 76]	<0.001
Valine	193 [174, 216]	229 [210, 250]	<0.001

Median [interquartile range].

The correlations between SSPG concentration and the 24 amino acids in the entire population are listed in Table3. There was a significant correlation between SSPG and 14 amino acid concentrations; all were positive, with the exception of glycine. The amino acids with the strongest correlation (*r* ≥ 0.4) were glutamic acid, isoleucine, leucine, and tyrosine. In view of the earlier reports (Felig et al. 1969; Newgard et al. 2009) of the association between increases in plasma amino acid concentrations and obesity, the correlation coefficients between individual amino acids and SSPG concentrations were adjusted for differences in BMI (Table3). The only impact of this adjustment was the loss of statistical significance between SSPG with three amino acids with *r* ≤ 0.2 (beta-alanine, lysine, and methionine); all of the other correlations between amino acid and SSPG concentration remained significant. The results were comparable when the correlations between SSPG and individual amino acids were adjusted for waist circumference versus BMI (Table3); only proline was no longer significantly associated with SSPG concentration. Both measures of adiposity were similarly associated with SSPG concentration (Waist, *r* = 0.53; BMI, *r* = 0.48, *P *< 0.001).

**Table 3:** Correlation (*r*-vale, 95% CI) with insulin resistance unadjusted and adjusted for measures of adiposity

Amino acids	Unadjusted	Adjusted for BMI (*n* = 180)	Adjusted for WC (*n* = 140)
Alanine	0.30 (0.18, 0.43)**	0.27 (0.14, 0.39)**	0.20 (0.03, 0.34)*
Arginine	0.11 (−0.02, 0.24)		
Asparagine	−0.03 (−0.17, 0.13)		
Aspartic acid	0.37 (0.25, 0.48)**	0.30 (0.16, 0.44)**	0.24 (0.08, 0.39)**
Beta-alanine	0.18 (0.03, 0.31)*	0.14 (0.003, 0.30)	0.17 (−0.02, 0.34)
Citrulline	−0.06 (−0.20, 0.08)		
Glutamic acid	0.44 (0.32, 0.55)**	0.32 (0.19, 0.45)**	0.25 (0.09, 0.40)**
Glutamine	−0.13 (−0.25, 0.02)		
Glycine	−0.34 (−0.46, −0.20)**	−0.25 (−0.40, −0.10)**	−0.20 (−0.39, −0.02)*
Histidine	0.11 (−0.03, 0.24)		
Hydroxyproline (*n* = 161)	0.29 (0.12, 0.44)**	0.25 (0.08, 0.39)**	0.27 (0.09, 0.43)**
Isoleucine	0.46 (0.33, 0.58)**	0.37 (0.24, 0.49)**	0.30 (0.13, 0.46)**
Leucine	0.40 (0.27, 0.53)**	0.36 (0.21, 0.49)**	0.27 (0.10, 0.44)**
Lysine	0.20 (0.07, 0.33)**	0.14 (−0.005, 0.27)	0.13 (−0.05, 0.31)
Methionine	0.18 (0.04, 0.32)*	0.13 (−0.01, 0.27)	0.04 (−0.13, 0.22)
Ornithine	0.10 (−0.05, 0.25)		
Phenylalanine	0.35 (0.23, 0.47)**	0.26 (0.13, 0.39)**	0.20 (0.05, 0.34)*
Proline	0.31 (0.16, 0.45)**	0.23 (0.08, 0.37)**	0.14 (−0.03, 0.30)
Sarcosine	0.09 (−0.04, 0.23)		
Serine	−0.14 (−0.29, 0.01)		
Threonine	−0.004 (−0.16, 0.15)		
Tryptophan	0.15 (0.003, 0.32)		
Tyrosine	0.48 (0.34, 0.59)**	0.30 (0.15, 0.43)**	0.21 (0.06, 0.38)*
Valine	0.37 (0.25, 0.50)**	0.28 (0.15, 0.41)**	0.23 (0.08, 0.39)**
Metabolic variables			
BMI	0.48 (0.37, 0.58)**	–	–
Waist circumference	0.53 (0.43, 0.63)**	–	–
Fasting glucose	0.38 (0.25, 0.50)**	0.32 (0.20, 0.45)**	0.32 (0.17, 0.46)**
Triglyceride	0.39 (0.26, 0.52)**	0.38 (0.25, 0.49)**	0.28 (0.12, 0.43)**
HDL-C	−0.48 (−0.58, −0.35)**	−0.42 (−0.54, −0.28)**	−0.39 (−0.53, −0.22)**
Insulin (*n* = 114)	0.74 (0.65, 0.80)**	0.61 (0.52, 0.71)**	0.62 (0.50, 0.72)**

* *P* < 0.05

** *P *≤ 0.01. Adjustment for BMI or waist circumference (WC) was made if unadjusted correlations were significant.

**Figure 1:**
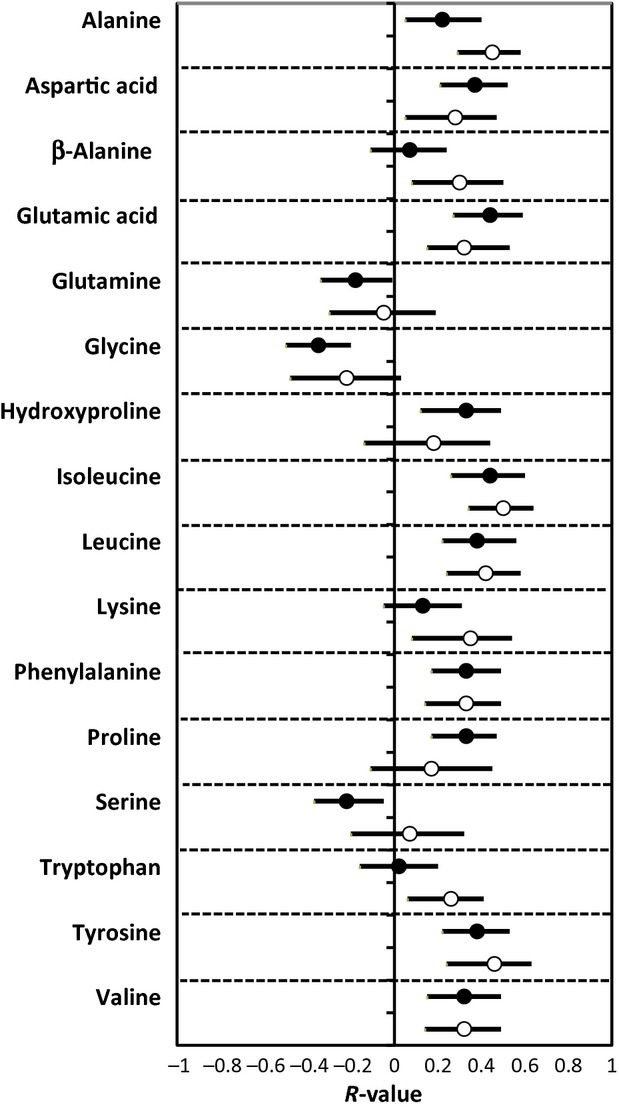
Relationship (*r*-value) between amino acids and insulin resistance in women (closed circle) and men (open circle). Error bars indicate 95% CI.

Figure1 shows the relationship between individual amino acids and SSPG concentration stratified by sex; only those amino acids significantly associated with SSPG concentration in one or both sexes are shown (Table4 lists *r*-values between all amino acids and SSPG concentration by sex). Although there was some variability in the strength of the relationship between SSPG concentration and individual amino acids in men versus women, there were no sex-specific significant differences in the strength of the correlations, with overlap in all confidence intervals (Fig.1, Table4).

**Table 4:** Correlation (*r*-value, 95% CI) with Insulin Resistance (SSPG) in Women and Men

	Women	Men
Amino acids		
Alanine	0.22 (0.05, 0.40)*	0.45 (0.29, 0.58)**
Arginine	0.08 (−0.06, 0.22)	0.19 (−0.07. 0.40)
Asparagine	−0.05 (−0.24, 0.15)	0.003 (−0.21, 0.22)
Aspartic Acid	0.37 (0.21, 0.52)**	0.28 (0.05, 0.47)*
Beta-Alanine	0.07 (−0.11, 0.24)	0.30 (0.08, 0.50)*
Citrulline	−0.07 (−0.24, 0.10)	−0.11 (−0.35, 0.14)
Glutamic Acid	0.44 (0.27, 0.59)**	0.32 (0.15, 0.53)**
Glutamine	−0.18 (−0.34, −0.01)*	−0.05 (−0.30, 0.19)
Glycine	−0.35 (−0.50, −0.20)**	−0.22 (−0.48, 0.03)
Histidine	0.07 (−0.09, 0.22)	0.17 (−0.08, 0.37)
Hydroxyproline	0.33 (0.12, 0.49)**	0.18 (−0.14. 0.44)
Isoleucine	0.44 (0.26, 0.60)**	0.50 (0.34, 0.64)**
Leucine	0.38 (0.22, 0.56)**	0.42 (0.24, 0.58)**
Lysine	0.13 (−0.05, 0.31)	0.35 (0.08, 0.54)**
Methionine	0.11 (−0.07, 0.28)	0.24 (0.03, 0.44)
Ornithine	0.08 (−0.11, 0.26)	0.07 (−0.20, 0.33)
Phenylalanine	0.33 (0.17, 0.49)**	0.33 (0.14, 0.49)**
Proline	0.33 (0.17, 0.47)**	0.17 (−0.11, 0.45)
Sarcosine	0.09 (−0.07, 0.23)	−0.02 (−0.26, 0.23)
Serine	−0.22 (−0.37, −0.05)*	0.07 (−0.20, 0.32)
Threonine	−0.02 (−0.20, 0.16)	0.04 (0.21, 0.27)
Tryptophan	0.02 (−0.16, 0.20)	0.26 (0.06, 0.41)*
Tyrosine	0.38 (0.22, 0.53)**	0.46 (0.24, 0.63)**
Valine	0.32 (0.15, 0.49)**	0.32 (0.14, 0.49)**
Metabolic variables		
BMI	0.50 (0.38, 0.62)**	0.38 (0.18, 0.55)**
Waist circumference	0.53 (0.39, 0.65)**	0.51 (0.31, 0.67)**
Fasting glucose	0.38 (0.20, 0.54)**	0.36 (0.17, 0.51)**
Triglyceride	0.38 (0.22, 0.54)**	0.37 (0.16, 0.55)*
HDL-C	−0.53 (−0.64, −0.39)**	−0.34 (−0.53, −0.14)**
Insulin	0.75 (0.66, 0.83)**	0.61 (0.46, 0.74)**

* *P* < 0.05

** *P* ≤ 0.01.

**Figure 2:**
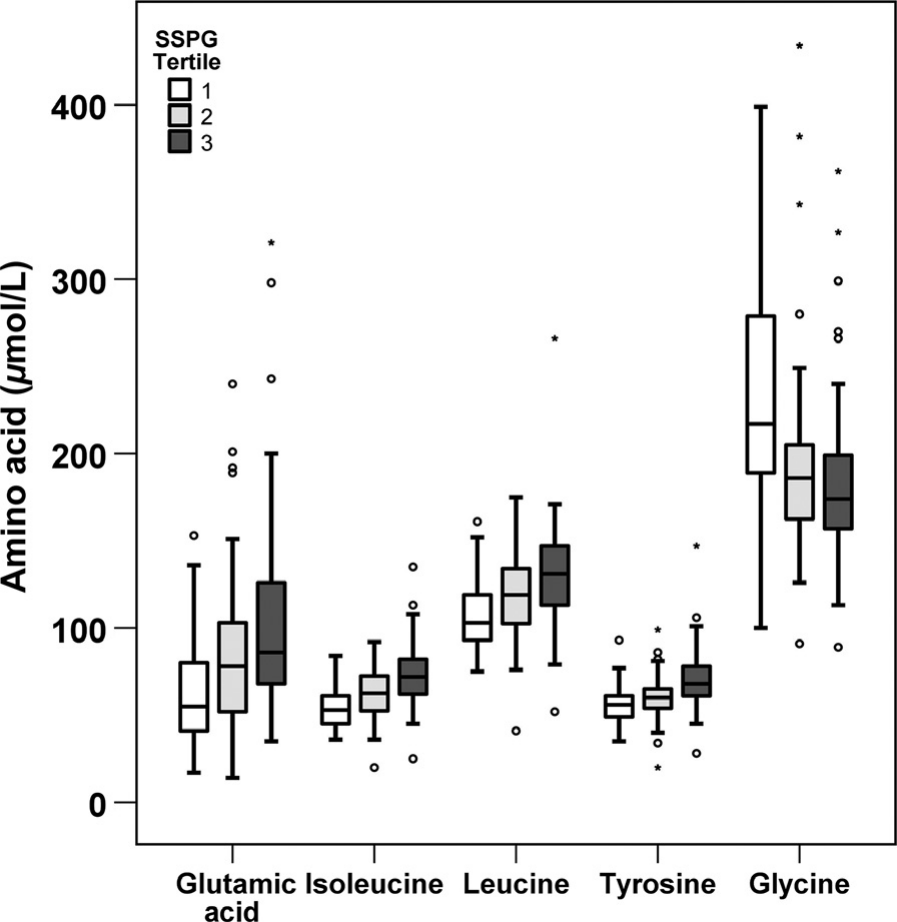
Boxplots of selected amino acids by tertile of SSPG concentration. Horizontal line within the box represents the median and boundaries of the box signify the lower and upper quartiles. Outliers are represented by 0 and *.

Finally, at the bottom of Table3 are seen the magnitude of the univariate correlations between SSPG concentration and the metabolites known to associate with insulin resistance. Fasting insulin had the strongest correlation with SSPG concentration, with an *r*-value of 0.74; a value substantially greater than the correlation between any amino acid and SSPG concentration. However, it should be noted that the magnitude of the correlation between SSPG concentration and four of the amino acids (glutamic acid, isoleucine, leucine, and tyrosine) was comparable to the relationship between SSPG and the other metabolites traditionally associated with insulin resistance (e.g., fasting glucose, triglyceride, HDL-C). Boxplots of amino acids with the strongest correlation with SSPG concentration and glycine are shown in Figure2 to illustrate differences in amino acid distribution by SSPG tertiles. Although there is overlap, mean amino acid concentrations differed significantly (*P *< 0.001) between those in SSPG tertile 3 (most insulin resistant) and SSPG tertile 1 (most insulin sensitive) for all illustrated amino acids (glutamic acid, isoleucine, leucine, tyrosine, and glycine).

## Discussion

The major goal of this study was to define the quantitative relationship between circulating amino acids and a direct measure of insulin resistance. Among the 24 amino acids evaluated, 13 were positively associated with SSPG concentration and glycine was negatively associated. Gall et al. (2010) also used a direct method to quantify insulin resistance (hyperinsulinemic, euglycemic clamp) in their study of the relationship between insulin resistance and selected amino acids, which were identified through nontargeted biochemical profiling. However, they only reported associations of insulin resistance with two amino acids, isoleucine and glycine. Similar to our study, the relationship between insulin resistance and the two amino acids was in the opposite direction; the more insulin resistant, the higher the plasma concentration of isoleucine and the lower the glycine.

In the most general sense, the amino acids we found to be most closely correlated with insulin resistance were the same ones identified in the early studies by Felig et al. (1969), and more recently by Newgard et al. (2009) to be elevated in the presence of obesity. Since obesity and insulin resistance are highly correlated (Kim et al. 2004), differentiating the relative importance of obesity versus insulin resistance in the pathogenesis of elevated plasma amino acid concentrations was another aim of our study. The fact that magnitude of the correlations between SSPG and amino acid concentrations remained when adjusted for BMI for majority of the amino acids supports the view that the relationship between insulin resistance and elevated plasma amino acid concentrations is independent of obesity. This conclusion is consistent with the findings of Tai et al. (2010), using the homeostasis model assessment of insulin resistance (HOMA-IR), to compare amino acid concentrations in “high HOMA” versus “low HOMA” groups.

Although in most instances the significant relationships between a given amino acid concentration and insulin resistance were positive, there was an inverse association between SSPG concentration and glycine. Glycine is a glucogenic amino acid, and it has been suggested that low levels may be secondary to increased hepatic gluconeogenesis in insulin-resistant states (Xie et al. 2013; Gall et al. 2010). However, this explanation alone does not explain why other glucogenic amino acids (e.g., alanine) are increased in insulin resistance. Glycine also participates in folate metabolism and glutathione biosynthesis, and may be consumed in these pathways (Gall et al. 2010).

An additional aim of our study was to compare the relative magnitude of the relationship between amino acid concentrations and insulin resistance to that between insulin resistance and associated metabolic variables. As shown in Table3, the correlations between SSPG concentration and plasma glucose, triglyceride, and HDL-C concentration were comparable to those between glutamic acid, isoleucine, leucine, and tyrosine concentrations. Whether the elevated amino acids contribute to the development of insulin resistance, are a consequence of the defect in insulin action, or simply associated biomarkers, are not addressed by our study, but the magnitude of the associations renders these questions worthy of further study. In that context, it has been argued that amino acids, especially BCAA, may play a pathogenic role in insulin resistance (Newgard et al. 2009; Newgard 2012).

The final issue addressed in this study was the role that sex differences might play in modulation of the relationship between circulating amino acids and insulin resistance. This issue has not received a great deal of attention, and the studies addressing it have relied on surrogate estimates of insulin resistance (Huffman et al. 2009; Würtz et al. 2012a,b, 2013). Our results indicate that despite comparable SSPG concentrations, men tended to have higher plasma amino acid concentrations than women, especially those amino acids associated with insulin resistance. For example, 10 out of the 14 amino acids that were higher in men were significantly associated with insulin resistance. Glycine was the only amino acid lower in men, and the only amino acid negatively associated with insulin resistance. Therefore, men had a more negative amino acid profile related to insulin resistance. However, the strength of association between SSPG and amino acid concentrations was generally similar between women and men (Fig.1). Although a less comprehensive list of amino acids were compared, Wurtz et al. also showed higher amino acid concentrations in men than women (Würtz et al. 2012a, 2013), except for glycine which was lower in men (Würtz et al. 2013). However, in contrast to our results, they found that the strength of associations between selected amino acids and HOMA-IR were different as a function of sex, being stronger in young men than women (Würtz et al. 2012a, 2013). A possible explanation for this discordance may be the differences in characteristics of the experimental populations. Thus, Wurtz et al. studied young (mean age 31 years), “metabolically healthy” individuals (Würtz et al. 2012a), while our population was older (mean age 52 years). We have previously shown that sex differences related to metabolic manifestations of insulin resistance are more magnified in younger than older individuals (Kim and Reaven 2013). In support of this explanation is the fact that the same group of authors reported no sex differences in middle-aged men and women (Würtz et al. 2012b).

There are limitations to our study. As this was a cross-sectional study, we have described quantitative associations between amino acids and insulin resistance, and can only speculate on the cause and effect relationship between these two variables. Additionally, we did not have dietary history to evaluate the relationship between protein intake and plasma amino acid concentrations. Tai et al. showed no difference in protein intake in nonobese Asian men separated into lower and upper tertiles of HOMA-IR, despite significantly different levels of BCAA (Tai et al. 2010). They and others (Chevalier et al. 2005) have suggested that increased BCAA in insulin-resistant states may reflect increased protein turnover and/or reduced rates of BCAA catabolism. Finally, although we have provided information that has heretofore not been available, our findings are based on measurements in only 182 individuals. However, we think that the information can serve usefully as the infrastructure with which to address more substantive questions, for example, the cause and effect relationships between circulating amino acids and insulin resistance, as well the potential clinical utility that amino acid concentrations might have as biomarkers to identify insulin-resistant individuals.

In conclusion, the results of our studies have provided for the first time the quantitative relationship between a direct measure of insulin resistance and the plasma concentrations of 24 amino acids in a population of apparently healthy individuals. In addition, we have shown that significant relationships exist between insulin resistance and 14 amino acids that are independent of obesity, and that the magnitude of the relationship between the concentrations of these amino acids and insulin resistance are comparable in men and women. Finally, the magnitude of the relationship between insulin resistance and several amino acids approaches that of several metabolic variables known to be associated with insulin resistance.

## Conflict of Interest

FH and MC are employed by Quest. The other authors have nothing to disclose.
